# A pilot study on AI-driven approaches for classification of mental health disorders

**DOI:** 10.3389/fnhum.2024.1376338

**Published:** 2024-04-10

**Authors:** Naman Dhariwal, Nidhi Sengupta, M. Madiajagan, Kiran Kumar Patro, P. Lalitha Kumari, Nagwan Abdel Samee, Ryszard Tadeusiewicz, Paweł Pławiak, Allam Jaya Prakash

**Affiliations:** ^1^School of Computer Science and Engineering, Vellore Institute of Technology, Vellore, Tamil Nadu, India; ^2^Department of ECE, Aditya Institute of Technology and Management (A), Tekkali, Andhra Pradesh, India; ^3^School of Computer Science and Engineering, Vellore Institute of Technology, Amaravati, Andhra Pradesh, India; ^4^Department of Information Technology, College of Computer and Information Sciences, Princess Nourah bint Abdulrahman University, Riyadh, Saudi Arabia; ^5^Department of Biocybernetics and Biomedical Engineering, AGH University of Science and Technology, Krakow, Poland; ^6^Department of Computer Science, Faculty of Computer Science and Telecommunications, Cracow University of Technology, Krakow, Poland; ^7^Institute of Theoretical and Applied Informatics, Polish Academy of Sciences, Gliwice, Poland

**Keywords:** artificial intelligence, mental disorder (disease), air pollution, smoking–adverse effects, noise pollution, alcohol

## Abstract

The increasing prevalence of mental disorders among youth worldwide is one of society's most pressing issues. The proposed methodology introduces an artificial intelligence-based approach for comprehending and analyzing the prevalence of neurological disorders. This work draws upon the analysis of the Cities Health Initiative dataset. It employs advanced machine learning and deep learning techniques, integrated with data science, statistics, optimization, and mathematical modeling, to correlate various lifestyle and environmental factors with the incidence of these mental disorders. In this work, a variety of machine learning and deep learning models with hyper-parameter tuning are utilized to forecast trends in the occurrence of mental disorders about lifestyle choices such as smoking and alcohol consumption, as well as environmental factors like air and noise pollution. Among these models, the convolutional neural network (CNN) architecture, termed as DNN1 in this paper, accurately predicts mental health occurrences relative to the population mean with a maximum accuracy of 99.79%. Among the machine learning models, the XGBoost technique yields an accuracy of 95.30%, with an area under the ROC curve of 0.9985, indicating robust training. The research also involves extracting feature importance scores for the XGBoost classifier, with Stroop test performance results attaining the highest importance score of 0.135. Attributes related to addiction, namely smoking and alcohol consumption, hold importance scores of 0.0273 and 0.0212, respectively. Statistical tests on the training models reveal that XGBoost performs best on the mean squared error and *R*-squared tests, achieving scores of 0.013356 and 0.946481, respectively. These statistical evaluations bolster the models' credibility and affirm the best-fit models' accuracy. The proposed research in the domains of mental health, addiction, and pollution stands to aid healthcare professionals in diagnosing and treating neurological disorders in both youth and adults promptly through the use of predictive models. Furthermore, it aims to provide valuable insights for policymakers in formulating new regulations on pollution and addiction.

## 1 Introduction

The mental health of an individual is considered one of the most crucial aspects of one's overall wellbeing. It is often related closely to the emotional, psychological, and social wellbeing of humans. Mental health refers to the wellbeing of a person that helps one to maintain their mental state and function well to realize their potential. It helps individuals to deal with their psychological stress and maintain a positive relationship with body and soul. Even physicians' mental health has also concerning for decades. For example, in 1936, four levels of impairment of the psychological functioning of medical students were identified, reflecting varying degrees of stress and challenges in their educational pursuits (Centers for Disease Control and Prevention, 2023). Nowadays, some representative scales are used to measure mental health, such as the Self-reporting Inventory (SCL-90), Minnesota Multiphasic Personality Inventory (MMPI), Self-Rating Anxiety Scale (SAS), Self-Rating Depression Scale (SDS), Eysenck Personality Questionnaire (EPQ), the Sixteen Personality Factor Questionnaire (16PF) (Kim et al., 2020). Mental health shapes a person's behaviors, thought processes, feelings, and much more throughout their life. It is a very important aspect that plays a significant role in enhancing the overall quality of life. As an example, the COVID-19 pandemic has had a great impact on people in various ways affecting their physical, psychological, and emotional wellbeing as well as social and economic problems (NIH, 2007; Sass et al., 2017; Helbich, 2018).

Emergencies and disasters are common, for example, due to severe weather events associated with climate change (such as hurricanes and floods), war-related or economic displacement, and terrorist attacks (Sass et al., 2017). Social media is also integral to the lives of teenagers, but adolescent voices need to understand the relationship between social media and mental health as it has a great impact of stress on them which can change their emotional and cognitive functions (Hammersen et al., 2016; Centers for Disease Control and Prevention, 2023)^1^. Mental health surrounds a wide range of disorders that can affect individuals in various physical and psychological ways. A growing number of UK and international studies show that mood disorders are on the rise among young people, particularly girls and young women (Gunnell et al., 2018). According to the National Health and Morbidity Survey (NHMS) 2017, one in five people in Malaysia is depressed, two in five people are anxious, and one in ten people is stressed (Srividya et al., 2018). Mental illness can sometimes be a group of disorders that affect the daily life of a person. A study by the Victorian Government provides valuable insights into several types of mental health disorders, some of which are as follows (Velten et al., 2014):

- Anxiety disorders: anxiety disorders are not one but a cluster of mental health issues like depression, social phobia, specific phobias, and panic attacks (Velten et al., 2014). A person suffering from anxiety disorders may suffer acute depression and panic attacks. According to the statistics provided, about 25% of the population suffers from anxiety disorder and requires professional medical treatment at one point in their lives. However, it is of great importance to understand that not all cases of anxiety attacks can be categorized as a disorder. Common symptoms of anxiety disorder, especially panic attacks, include dizziness, shortness of breath, increase in pulse rate, nausea, choking feeling, and several others. A person is diagnosed with generalized anxiety disorder if the symptoms last more than a period of six months or if the person is unable to control themselves when they suffer from any of the symptoms. Treatments like cognitive behavioral therapy, exposure therapy, anxiety management and relaxation techniques, and medication like antidepressants and benzodiazepines help in recovery from anxiety disorders.
- Bipolar affective disorders and depression: bipolar disorder, formerly known as manic depression, involves an individual experiencing extreme cases of low and high moods, referred to as manic or hypomanic (Geršak et al., 2012). When a person is suffering from acute depression and has depressive episodes, they can be diagnosed with bipolar affective disorder. The disorder may be induced genetically in 80% of the cases or from factors such as brain chemicals, environmental factors, physical illness, and stress. Symptoms begin in the early adulting years. There are several types of bipolar disorder, namely bipolar-I disorder, bipolar-II disorder, cyclothymic disorder, and mixed episodes. Bipolar disorder requires long-term treatments that include mood-stabilizing medications, antidepressants, antipsychotic medications, psychological therapies, and many more.
- Eating disorders: one of the most severe mental illnesses that affect people of all ages and from different walks of life. According to the study, the count of affected Australians is expected to be approximately one million (Velten et al., 2014). When a person either doesn't feel like eating or, inversely, indulges in binge eating, then the person is known to suffer from eating disorders. Anorexia nervosa, bulimia nervosa, and binge eating are a few types of eating disorders. The disorder may be induced via environmental, psychological, and biological factors and can be identified from symptoms like sudden weight fluctuations, faintness, fatigue, anxiety, sensitivity to cold, and several others. Seeking guidance from a medical professional is best advised in cases of eating disorders and may be treated via medication.
- Post-traumatic stress disorders (PTSD): PTSD refers to a set of reactions developed in a person who has suffered from a traumatic event that may have threatened their life. Events like accidents, physical, mental, or sexual assaults, torture, etc., are the most common reasons that may trigger PTSD. It is often associated with childhood traumas that affect a person's mental health throughout their lives. The common PTSD symptoms involve extreme heart palpitations or panic attacks when the victim relives the traumatic situation through recurrence as a memory or flashback, showing detachment when a certain topic comes up in conversations, using a negative adjective to describe oneself, for example, saying, “I'm a bad person”, and being overly alert and have sleep difficulties. PTSD may trigger defiant behavior, attention deficit hyperactivity disorder (ADHD), or depression in adolescents. On the other hand, it may lead to substance abuse or other addictions in later years of life. Treatments involve first undergoing trauma-focused therapy and later taking antidepressants or other medications as prescribed by the concerned doctor.

Mental illness is all about the change of brain structure, and its function and related to biological basis. The probability of having a mental disorder can be increased by environmental variables, including lead exposure and cigarette smoking, as well as brain trauma, poor nutrition, and exposure to chemicals (De Figueiredo et al., 2021). A mental health diagnosis involves many steps, and this is not one simple process. Diagnosis begins with specially designed questions about symptoms and medical history and sometimes an execution physical examination (Murphy, 2012). Lifestyle choices and habits play a vital role in maintaining an individual's mental health. Up to 22% of the global burden of disease and 23% of deaths are attributable to environmental pollution; the general public is inevitably exposed to environmental pollutants (De Figueiredo et al., 2021). Habits like meditation and regular exercise are related to better mental health, however, addictions like smoking, alcohol consumption, chewing tobacco, and use of narcotics and drugs induce harmful effects on a person's mental health. As per the World Health Organization (WHO) (Slavin, 2016) records, more than 8 million people are killed by tobacco each year, including 1.3 million non-smokers who are exposed to secondary smoking. In another study by the Center for Disease Control and Prevention (Gunnell et al., 2018), it is discussed that compared to those without mental health disorders, adults with mental health conditions smoke substantially more frequently.

In 2020, smoking cigarettes within the previous month was reported by 23.10% of individuals in America with any type of mental illness. Roughly a quarter of individuals in the U.S.A. experience some form of mental disorder, comprising 25% of the population. Interestingly, this demographic, constituting 25%, contributes to around 40% of smoking consumption. A study by the Mental Health Organization (Xu et al., 2018) reveals that alcohol is a depressant that affects your feelings, thoughts, and behavior by disrupting the balance of the neurotransmitters in your brain. Over time, alcohol causes a decline in the number of neurotransmitters in our brains, which leads to many mental disorders like anxiety, depression, and many more. These claims are also substantiated by the WHO (Slavin, 2016). The use of drugs on the other hand, is much more harmful to the body and mind. Consumption of ill-prescribed or illegal narcotics may lead to serious irreversible mental health conditions like autism, depression, etc. Though drugs may help in pain relief in medical uses under supervision, however, long usage may lead to addiction and potential loss of health and mental stability (Danese et al., 2020). Thus, it is revealed that addictions like smoking and alcoholism have a direct impact on mental health.

Environmental pollution is not a new phenomenon, but it is one of the world's greatest problems that mankind is facing and a major environmental cause of illness and death (O'reilly, 2020). In 1991, the American economists Grossman and Krueger introduced the Kuznets curve into studies of the relationship between economic growth and environmental pollution (Romeo, 2017). Air is polluted when some unwanted substance is present in the atmosphere that changes its purity. The pollutants can be natural or man-made. Some substances that contaminate the air such as particulate matter (PM), nitrogen dioxide (*NO*_2_), sulfur dioxide (*SO*_2_), carbon monoxide (*CO*), ozone (*O*_3_) etc (Xu et al., 2018). Gaseous components and particulate matter (PM) cause ambient urban air pollution. The former include ozone (*O*_3_), volatile organic compounds (VOCs), carbon monoxide (CO), and nitrogen oxides (*NO*_*x*_) are well-established as inflammatory stimuli in the respiratory tract (Ukaogo et al., 2020). Heavy metals such as lead, when absorbed into the human body, can lead to direct poisoning or chronic intoxication, depending on exposure. Particulate matter 2.5 (PM 2.5) means small airborne particles with a diameter of 2.5 micrometers that penetrate the respiratory system via inhalation, causing respiratory and cardiovascular diseases, reproductive and central nervous system dysfunctions, and cancer (Liang et al., 2019). Environmental factors such as air pollution and green space have raised much attention in the world, however, the use of such factors in mental health analysis remains debatable. Pollution, working conditions, weather conditions, etc., are physical factors, while stimulation, messy environment, and lack of green outdoors are other factors that may lead to mental illness (Glencross et al., 2020). Current research suggests that people are reluctant to go outdoors due to air pollution (Xu et al., 2018). Noise pollution refers to the unwanted sounds that hamper the acoustic environment of nature and cause a negative impact on physical health as well as mental health. It happens when the sound level exceeds the comfort level and ends up in stress, irritation, annoyance, and mental illness. Noise pollution (Manisalidis et al., 2020) can arise from various sources like road traffic, aircraft, industrial processes, construction, entertainment events, and everyday household work. Factors like demographics, chronic illness, and social support influence people who are more likely to suffer from mental illness than general people, both men and women. Except for air traffic, all other noises have a serious impact on mental health.

The mental health of young people has been significantly declining due to the advent of social media culture, virtual reality, augmented reality, and longer screen time. Metropolitan cities are overrun by air and noise pollution, which further increases the severity of mental health deterioration. Smoking and alcoholism have also become rapidly popular among youth, and the devastating effects can be seen in the mental health surveys. This research is an analysis of the situation and extent to which the youth's mental health is affected by environmental and addiction-based factors. This study will prove to be useful for scientists, researchers, psychiatrists, counselors and doctors to understand the extent of neurological disorders in the individual and give them appropriate treatment. This work uses innovative approaches to extract some important insights that will help society comprehend mental health and its relationship to lifestyle, environmental, and addiction-based issues. The major key contributions of the study are as follows:

- The authors presented the analysis of reliable methodologies with various machine and deep learning for the detection of mental disorders in individuals.
- The authors utilized various environmental attributes such as air pollution, noise pollution, and lifestyle factors like smoking, alcoholism and drug usage for the prediction of mental health.
- The investigation utilized a survey-based dataset provided by the Cities-Health Initiative^2^, exploring various AI-based techniques for predicting mental health conditions.
- Results from the Stroop test (Qiao, 2020) were incorporated to enhance the accuracy of predicting mental disorders by identifying additional predictive factors.

The dataset consists of self-examined answers to a questionnaire that focuses on the numeric score rather than the specific type of mental disorder experienced. Therefore, the number of mental health diseases reported by individuals should not be considered medically accurate. However, the trend can be evaluated as a population by comparing the mean values. The research suggests that an individual's mental health may be higher or worse than the population average. The paper, thus, aims to predict with high accuracy the extent to which an individual is suffering from neurological disorders as compared to the population's statistical mean. The paper does not comment on the exact mental disorder experienced.

The remaining portion of the manuscript is organized as follows: The detailed literature survey related to mental health care is discussed in Section 2. The database utilized in this work is mentioned in 3. The proposed methodology for the detection of mental health care is in Section 4. Experimental results and discussion of the different methodologies are explained in Section 5, and finally, the outcomes of the manuscript are concluded in Section 6.

## 2 Literature survey

In the past, different researchers have focused on mental diseases, and several studies used AI-based algorithms to evaluate massive datasets and predict mental health prospects. Sass et al. (2017) have tried to find a connection between air pollution exposure and mental anguish based on nationally representative panel data from the United States. They observed an annual average measure of air pollution levels in respondents' residential census blocks between 1999 and 2011, and the study noticed that a PM 2.5 increase may increase psychological distress. The findings suggest that humans must pay attention to the psychological distress induced by air pollution to reduce both the personal and social costs of mental health. Kessler 6 (K6) score is the measurement of psychological distress, it is a dependent variable and a composite instrument of six items assessing how an individual feels sad, nervous, hopeless, worthless, restless, or everything was an effort during the past 30 days. It can be observed that the K6 score was the worst among respondents and at that time, PM 2.5 was with higher concentrations. This describes every 5-unit change in PM 2.5, the K6 score can be changed. The study of Model-1 quantifies the relationship between air pollution and psychological stress where coefficient estimates were used which define the 5-unit change in K6 score as related to the change in PM 2.5. In the work, authors observed that the result in white women is very noticeable while using a bivalent version of K6 a significant positive result was obtained. This indicates that both gender and racial-ethnic characteristics should be taken into account when observing the association between air pollution and psychological distress (Xu et al., 2018).

The long-term effects of air pollution on mental health have been studied in Indonesia by Kim et al. (2020). Experimenters found that exposure to fire in 1997 affected people even after 10 years, and they are clinically proven depressed. They claimed education, economic status, and marriage to be the moderators for the negative effects. The author has evaluated the Center for Epidemiological Studies Depression Scale (CES-D) and on a scale of 0–30, if a person has a higher score, it represents severe depression. Participants calculated some special variables like PM 2.5, and it was tested separately in men and women. Ordinary Least Squares (OLS) Regression was used to calculate the correlation between the PM 2.5 score and the depression score. To make sure of the evaluation, researchers executed additional analysis using ordered probit and ordered logit regression. These were used for ordinal dependent variables like depression scores, and similar results were found. The findings were agreed on different methods. Studies found that men are more blissful than women. Surprisingly the affected people were women, and it is said that maybe men recovered fast within 10 years, but women couldn't. Even after analyzing the long-term effects of air pollution on mental health, it suggests that the short-term effects are even worse (Sumathi and Poorna, 2016).

Hammersen et al. (2016) expressed the effects of noise pollution on mental health, especially cardiovascular effects. The study aimed to survey the relationship between individual levels of noise annoyance caused by noise from different sources in the living environment of German adults in 2012. This research suggests a relationship between high noise annoyance and mental health issues. Researchers found a higher frequency of mental health in women than men. In each group of four women, where all were disturbed by overall noise, more than one woman reported mental health impairment. But in a group of ten people who are not disturbed by noise, only one woman is mentally impaired. Men also went through the same survey but had smaller portions of mental health impairment than women and less annoyance level overall. The *p*-values were derived from Pearson's χ^2^-test for a two-way table between the noise annoyance variable and the mental health variable by sex and noise source to examine statistically significant differences. Logistic regression models were used to calculate odds ratios (OR) of impaired mental health with 95% Confidence Intervals (CIs) and *p*-values by noise annoyance level and noise sources, adjusted for potential covariates which were added to the model step by step. In the first model, socio-demographics such as age, residential area, and socio-economic status were included. Chronic disease was added in the second model and social support was included in the third model. When the *p*-value was < 0.05, the result was considered meaningful. Partially outcomes increased for slightly moderately annoyed people than not-annoyed people, but in the final model, chronic disease and social support reduced the odds ratio, which moderated the result of slightly moderately annoyed people subgroups. Nevertheless, in the final model, both men and women who are highly annoyed (HA) by noise overall were more than twice as likely to have impaired mental health (Manisalidis et al., 2020).

In 2018, Srividya et al. (2018) presented a study of using behavioral modeling in cases of mental disorders using machine learning (ML) techniques on the questionnaire-based dataset. In the study, the authors used numerous variables, such as narcotics and drug addiction, that contribute to mental health issues and result in mental disease. The researchers identified the benefits of using ML and AI in terms of computational powers and, thus, used several models such as Support vector machines (SVM), Decision trees (DT), Naïve Bayes classifier (NBC), K-nearest neighbor (KNN) classifier and Logistic regression. In the beginning, unsupervised learning techniques were used for the responses gathered from the target group for the constructed questionnaire. To verify the labels that clustering generated, the mean opinion score was computed. Then, classifiers were developed to predict a person's mental health using these cluster labels. The best accuracy of 90% was achieved using the ensemble bagging techniques models and the random forest model, however, NBC had the lowest accuracy at 73% and the accuracies reported by the other classifiers were reasonable.

Cho et al. (2019) presented a review study in 2019, and they focused on applying ML algorithms to detect mental illness and provided some recommendations for practical applications of these methods. In the methodology, authors used five standard ML algorithms SVM, Gradient Boosting Machine (GBM), Random Forest, NBC, and KNN, for the prediction of mental health. The results were effectively organized by the type of disorder analyzed, such as depression, autism spectrum disorders, PTSD, and schizophrenia. In the results of schizophrenia, the authors revealed that the SVM model's average discrimination accuracy between people with schizophrenia and healthy controls was 90%, while its average accuracy in differentiating between those with schizophrenia and bipolar illness was 88%. Whereas, SVM performed sub-optimal with only 77% accuracy in the cases of autism, where NBC achieved a maximum accuracy of 80%. The majority of the SVM classifiers developed in the studies demonstrated more than 75% high accuracy, as was predicted. The ensemble techniques of GBM and Random Forest were also employed as the main algorithms to categorize specific mental health patients because of their benefits in processing several data sets concurrently without feature selection.

Velten et al. (2014) presented a comprehensive study about the multi-faceted relationship between life choices and mental health. In the study, lifestyle parameters such as alcohol use, smoking, body mass index, and social and circadian regularity were evaluated by self-report. However, other criteria include the amount of time spent engaging in physical and mental activities. The parameters like Depression, anxiety, stress, and life satisfaction factors were associated with the outcomes. During analysis of the questionnaire-generated data, it was revealed that women reported engaging in mental and cultural activities more frequently than males, but men reported drinking alcohol more frequently. Also, moderate drinkers had better mental health than abstainers and highly frequent drinkers, according to the quadratic term of alcohol frequency, which was negatively connected with life satisfaction and positively correlated with levels of sadness and anxiety. Abstainers and heavy drinkers had worse levels of mental health in all four outcome variables, namely life satisfaction, depression, anxiety, and stress, according to the significant squared factors of alcohol consumption frequency. By observing the results on smoking as a factor, we noticed that regarding all four end measures, higher mental health was linked to more frequent mental/cultural activities, abstinence from smoking, regular social interactions, and circadian rhythms. Thus, the study, in many statistical ways, proves the ill effects of smoking and alcohol on one's mental health, and in doing so, gives a high correlation between life choices and mental disorders.

Therefore, after a close study of the literature on the prediction of disease and mental disorders, the need to use ML algorithms is identified, and references to the various ML models used in the research field are studied. Classical ML techniques like SVM and KNN perform well, however, more complex deep learning models (DL) must also be explored for predictions (Allam et al., 2020; Prakash, 2022; Sahoo et al., 2022; Venkata Phanikrishna et al., 2023).

## 3 Materials

The dataset ^2^ provides comprehensive information about the unique perception of the impact of air pollution on human health. It consists of data collected for the Barcelona epidemiological research study within the framework of the CitieS-Health project. This dataset is being utilized for the first time to analyze Barcelona residents' mental health. The dataset was created during the COVID-19 pandemic and thus contains vital insights on mental health, addiction, pollution, and the environment. Data were collected from September 2020 to March 2021 in Barcelona (Spain) and the interrelation between short-term exposure to air pollution and mental health. CitieS-Health aimed to set citizens' concerns at the center of the environmental epidemiology research agenda by addressing health issues of concern to them. The proposed novel analysis of this dataset using AI models will also give insights into the effect of the “lockdown' phase on adult mental health. The Stroop test is a neuropsychological test that evaluates an individual's cognitive interference and ability to inhibit automatic responses. Researchers (Qiao, 2020) presented an attempt to evaluate the most used color-word conflict test; different versions of the Stroop test define different levels of psychophysiological results. The Stroop test is being evaluated with some steps such as response times, Stroop effect calculation, accuracy, individual differences, group comparisons, neuroimaging studies, and Stroop test variations. There are some theories (Pintelas et al., 2018) for the Stroop effect:

- **Selective attention theory:** It determines what information is being processed and what is being ignored. In that case, identifying word color is more important than reading.
- **Automaticity theory:** In this case, readers give more importance to actual reading than identifying the colors.
- **Speed of processing theory:** This makes color identification tough after reading the text due to processing speed.
- **Parallel distributed processing:** This theory says that the brain creates different pathways for different tasks.

The dataset contains 95 columns and 3,349 rows. The attributes in the dataset are namely mentalhealth_survey, physical_activity, drugs, alcohol, computer_use, diet, illness, drink, stroop_test, etc., and these are all categorical data. The dataset has numerical data as well, such as year, month, day, hour, occurrence_mental, energy, stress, wellbeing, sleep quality, z_performance, mean_congruent, no2bcn_24, pm25bcn, sec_noise65_day, pressure_24h, etc. Categorical data are mostly lifestyle choices, and numerical data are mostly responsible for air pollution.

## 4 Proposed methodology

**Figure 1:**
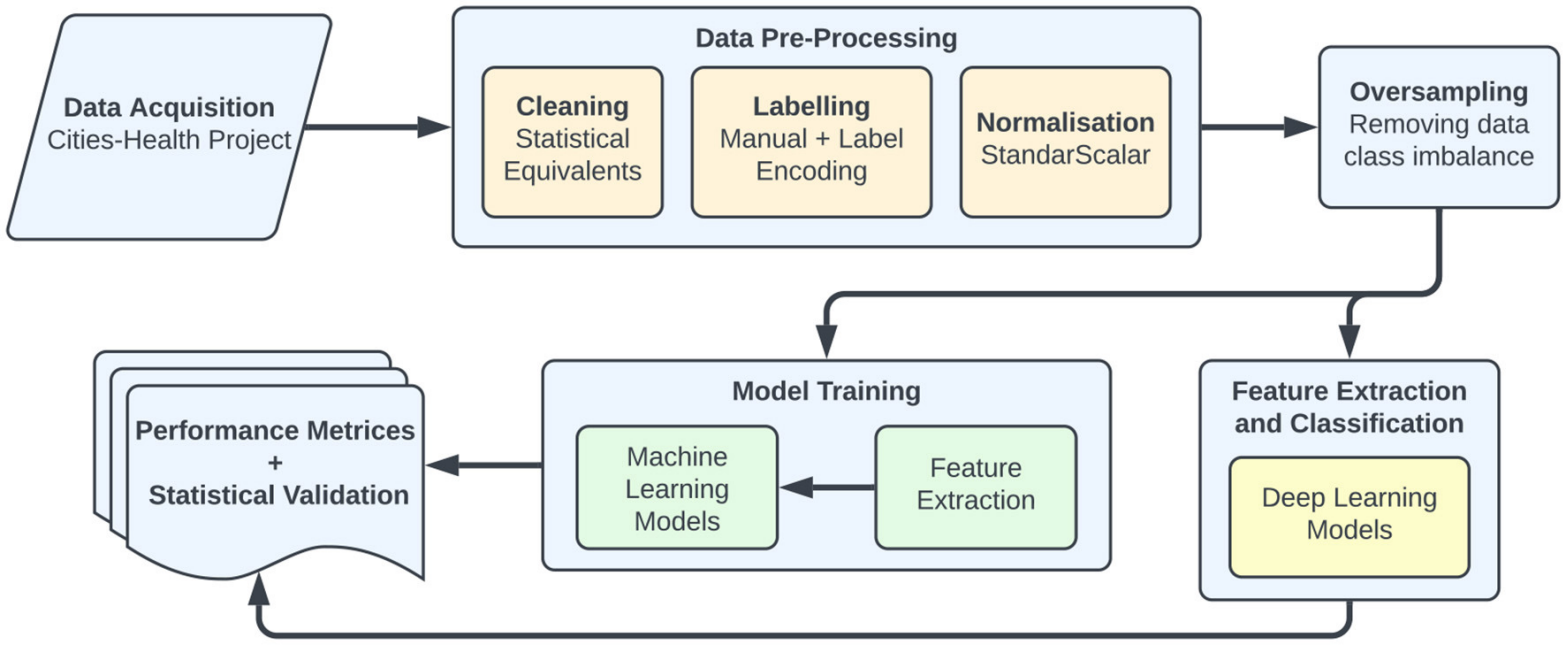
Workflow of the methodologies utilized in this work to detect mental health care.

The overall block diagram of the proposed method for the prediction of mental health diseases is shown in Figure 1. The suggested system consists of five key stages: pre-processing, oversampling, model development and training, tuning, and detection.

### 4.1 Data pre-processing

The data pre-processing is the initial step in the proposed approach and the dataset was translated into English as the original dataset was in Spanish, given the source as aforementioned. Columns like gender, alcohol, drugs, etc., were not only labeled in Spanish but also contained values in Spanish. The translated dataset was then subjected to further processes. Moreover, dimensionality reduction is used in machine learning which is effective in removing irrelevant and redundant data, increasing learning accuracy, and improving result comprehensibility. However, the recent increase in the dimensionality of data poses a serious challenge to many existing feature selection and feature extraction methods in terms of performance and effectiveness (Wang et al., 2021). The obtained dataset has a few deficiencies and challenges, which were dealt with using the techniques mentioned in the following stages.

#### 4.1.1 Cleaning

Cleaning a dataset is the process of removing inconsistencies from the dataset. To thoroughly examine the data, the research utilized the Python package pandas and its built-in techniques like “isnull()” and “describe()”. The results showed that the original dataset contained missing values, probable outliers, and categorical data. The first step was to remove the unnecessary and out-of-scope columns like patient ID, date of survey, and many more. The original dimensionality of 95 was shrunk to 29 after the complete removal of some columns or by using techniques like principal component analysis (PCA) to merge various mutually exclusive columns as one. This reduced dimensionality has helped in better computational performance while training and testing the models. Also, the presence of too many irrelevant attributes might have caused problems like over-fitting and induction of bias. To handle missing data, the mean of numeric values of columns is computed, and the missing values of that column are filled using the mean value. A similar technique was used to fill the columns with categorical data, however, rather than the mean, the median was used in this case. The dataset contained duplicate columns where the values of one column were multiplied by a factor of 30 and placed in a separate column. Such columns with multiples are deleted after identification to remove bias and avoid inducing effects on training results. Though restraining from deleting rows to retain population, only the rows with extremely high missing values are deleted to avoid further imbalance and bias.

#### 4.1.2 Labeling

The dataset, now with no missing values, contains both numerical and categorical data in the columns. Therefore, to convert the categorical data values into numeric values, manual labeling was used for the data values. Throughout the dataset, “yes” was encoded as 1, and “no' was encoded as 0. Similar trends are followed for “female” and “male” in the gender section. Techniques like one-hot encoding and label encoding are discussed; however, due to the small number of unique values per column, manual labeling is the best solution. The target attribute, “mental_occourance” contained values ranging from 0 to 14. The research predicts the relation of various environmental and lifestyle factors with mental health occurrences as below or above average in the population. It is predicted that an individual with a given set of constraints would have fewer mental health occurrences less or more as compared to the average. To do so, the mean of the target attribute was calculated as 6.25. The values below the mean value that is 1–6 were encoded as 0, and values above the mean were encoded as 1 to facilitate the aim of the research.

#### 4.1.3 Normalization

The numeric columns had large ranges in their values. To get fast convergence and numerical stability in the models it is decided to normalize the dataset using the library SKlearn and its inbuilt function “StandardScaler()”. The main reason behind choosing this methodology is that it gives the resultant numerical values in each column in such a way that the mean of all the values comes out to be 0, and the standard deviation becomes 1. Thus, this facilitates faster computation and avoids any chances of bias due to extremities in value ranges. All columns are normalized, including the ones with binary data values as well to maintain uniformity.

### 4.2 Oversampling

The dataset that is now normalized contains class imbalance, which is found using techniques like calculating class imbalance ratio and visualizing imbalance. A class imbalance is present when two classes in a binary classification problem have significantly differing amounts of instances. This mismatch may lead to biased and inaccurate model performance since ML algorithms have a propensity to prefer the majority class because of its larger representation. Several automated techniques that create synthetic data with similar values to the ones present in the minority class are discussed. However, the method of random oversampling is given privilege due to its accuracy in data values which may not have been achieved using techniques like synthetic minority over-sampling technique (SMOTE). In random oversampling, few data points from the minority class are duplicated in the dataset in such a way that the distribution of the new minority class still maintains the internal class balance and no new outliers are generated. The target attribute “mental_occourance”, as aforementioned, contained 1 as a minority class, and thus, selective data points at random are taken and duplicated in the dataset, with high precaution to avoid induction of bias and avoid imbalance.

### 4.3 Classification of the mental healthcare problems using AI models

The dataset is successfully processed through the previously described preparation and oversampling stages, resulting in a balanced dataset with no missing values, the fewest possible outliers, all normalized numerical columns, and a size reduction to 29. Furthermore, the processed data is forwarded to sophisticated artificial intelligence prediction models for simultaneous analysis of mental disorders using ML and DL models.

#### 4.3.1 Machine learning models

This proposed research has used eight ML models such as Logistic regression, Decision tree, Random Forest, Gradient boosting, Support vector machine (SVM), *k*-nearest neighbors (kNN), XGBoost, and LightGBM (Dhariwal et al., 2023). These models are used because of interpretability, data complexity, ensemble accuracy, and data size. Logistic regression is used for simple, interpretable, and effective binary classification tasks. It can handle binary tasks as well as approximate probabilities. Decision trees are easy to understand and can capture complex data, are also suitable for interoperability, and can handle both numerical and categorical data. Random forest is generally suitable for classification and regression tasks and it reduces over-fitting problems and gives the highest accuracy. Gradient boosting is a powerful machine learning technique that involves the iterative combination of weak learners on various data subsets, ultimately creating strong predictive models; notably, its ability to handle large datasets makes it particularly effective in diverse applications.

SVM is suitable for both linear and non-linear data. KNN can be used in small datasets and is very simple and flexible. XGBoost and LightGBM are mainly used for efficiency, handling large datasets, regularization to prevent over-fitting, handling missing data, tuning capabilities, flexibility, and high predictive accuracy. All the ML models were trained with the *k*-fold value of 4. These *k*-values of the *k* folds have been arrived at by training the models starting from *k* = 2 and incrementing the value by 1 until a decrease in the training accuracy, training quality, or statistical tests is observed. Thus, these values of *k* are the most optimum, as any value more than these would lead to high bias and poor performance as the model would have evaluated (seen) almost all the data during training. The main aim of the *k*-value selection is to optimize the trade-off between bias and variance of each model, thus different values are obtained for different models.

#### 4.3.2 Deep learning models

Deep learning is an effective research area in machine learning techniques and pattern classification association (Banna et al., 2023). In addition, four DL models were employed in this research (Dhariwal et al., 2023) such as Convolutional Neural Network (CNN) (Patro et al., 2022), Deep Neural Network (DNN) (Prakash et al., 2022), Recurrent Neural Network (RNN) (Allam et al., 2023), and Artificial Neural Network (ANN). In the paper, all the deep learning models CNN, DNN, RNN, and ANN are mentioned as DNN1, DNN2, DNN3, and DNN4, respectively. According to the benefits they offer in predictions and accuracy, each of the four models was picked. Because of its great accuracy in feature extraction and pattern identification, CNNs though frequently employed for image classification, produce outstanding results in textual datasets as well. DNNs are used for the research because they are more successful in precisely predicting outcomes by extracting key attributes. The ideal method for analyzing the temporal relationships in data is to use RNNs, which adds another perspective to the prediction analysis. Since ANNs are among the traditional DL models, it is only fair to compare the accuracy of contemporary models with that of the conventional model, which is regarded as one of the best in-textual prediction situations. Because Tensorflow, Keras, and PyTorch offer high-quality APIs that enable training and testing these models, the research makes use of their pre-defined neural network architectures in a Python environment. The basis of these DL models is, in a broad sense, the usage of multiple layers, termed input, hidden and output layers, that, with the use of nodes and weighted edges, help create a model that predicts the output for a dataset. The DL models use the “binary_crossentropy” loss function (Juliet et al., 2023) for training the models. This loss function is widely used in binary classification scenarios similar to the one in this research. Usually denoted as *L*(*y, p*), the loss function quantifies the dissimilarity between the actual binary labels or the true label (*y*) and the predicted probabilities that the data point lies within the class rightfully (*p*). The binary cross-entropy for each datapoint *i* from 1 to *N* (total number of data points) is given as *L*(*y*_*i*_, *p*_*i*_), where


(1) $$\begin{array}{l} L\left(y_{i},p_{i}\right)=-\left[y_{i}log\left(p_{i}\right)+\left(1-y_{i}\right)log\left(1-p_{i}\right)\right] \end{array}$$

Further, the average or summation of the individual losses for adding data points *i* from 1 to *N* is given as


(2) $$\begin{array}{l} L_{total}=\left(1/N\right)\overset{N}{\underset{i=1}{\sum }}L\left(y_{i},p_{i}\right) \end{array}$$

Therefore, the *L*_*total*_ represents the total loss for the entire dataset, and the model must be trained to minimize the overall loss.

### 4.4 Model tuning

Parameter tuning was performed by experimenting with different values of parameters like epochs, batch sizes and random state values in the case of each DL model separately until the most appropriate tuning was achieved. For ML models, the parameters about each model, like “max_depth”, “bootstrap”, “max_iter”, and others, are specific to each model, influencing aspects like tree depth, sampling strategy, and iteration limits, allowing fine-tuning for optimal performance. All the DL models are subjected to *k*-fold cross-validation and hyperparameter tuning, the values of which have been tabulated in Tables 1, 2, respectively. The major benefit of using specially hyperparameter-tuned models is the higher accuracy of the trained models for the specific dataset. Thus, Table 2 summarizes the hyperparameters and layer descriptions of each of the deep learning models in detail to elaborate the models' structure and architecture. The DNN1 model employs eight layers in it's architecture, followed by DNN2, DNN3 and DNN4 models employing three layers each in their respective architectures. The details of the internal structure of each layer of the four neural networks employed in this work have been tabulated in the order of their application in Table 2. The DL models used the “Adam” optimizers and the “ReLU” as the activation function. However, the output layers on all models used the “Sigmoid” function. Model fitting parameters for the DL models have been listed in Table 3. These parameters have been finalized after experimenting with various possible parameter values and taking the ones that result in maximum optimization of the final result.

**Table 1:** *k* values used in *k*-folds cross-validation for the deep learning models.

**S. No**.	**Classifier**	**Folds (*k*)**
1	DNN1	12
2	DNN2	10
3	DNN3	8
4	DNN4	12

**Table 2:** Layer description and hyper parameters for the deep learning models.

**Model**	**Layer mame**	**Hyperparameters**
DNN1	Convolutional 1	Filters: 128, Kernel Size: 3, Activation: ReLU, Input Shape: (X_scaled.shape[1], 1)
	MaxPooling 1	Pool Size: 2
	Convolutional 2	Filters: 64, Kernel Size: 3, Activation: ReLU
	MaxPooling 2	Pool Size: 2
	Flatten	No hyperparameters
	Dense 1	Neurons: 128, Activation: ReLU
	Dropout	Dropout Rate: 0.3
	Dense 2 (Output)	Neurons: 1, Activation: Sigmoid
DNN2	Dense 1	Neurons: 128, Activation: ReLU
	Dense 2	Neurons: 64, Activation: ReLU
	Dense 3 (Output)	Neurons: 1, Activation: Sigmoid
DNN3	SimpleRNN	Units: 128, Activation: ReLU, Input Shape: (X_scaled.shape[1], 1)
	Dense 1	Neurons: 32, Activation: ReLU
	Dense 2 (Output)	Neurons: 1, Activation: Sigmoid
DNN4	Dense 1	Neurons: 64, Activation: ReLU, Input Dimension: X_scaled.shape[1]
	Dense 2	Neurons: 32, Activation: ReLU
	Dense 3 (Output)	Neurons: 1, Activation: Sigmoid

**Table 3:** Hyper-parameters used for DL Models after tuning.

**Deep learning models**	
**Parameter**	**Value**
Epochs	0 – 100
Batch size	32 – 64 – 128
Validation split	0.1 – 0.2
Test size	0.15 – 0.2

**Figure 2:**
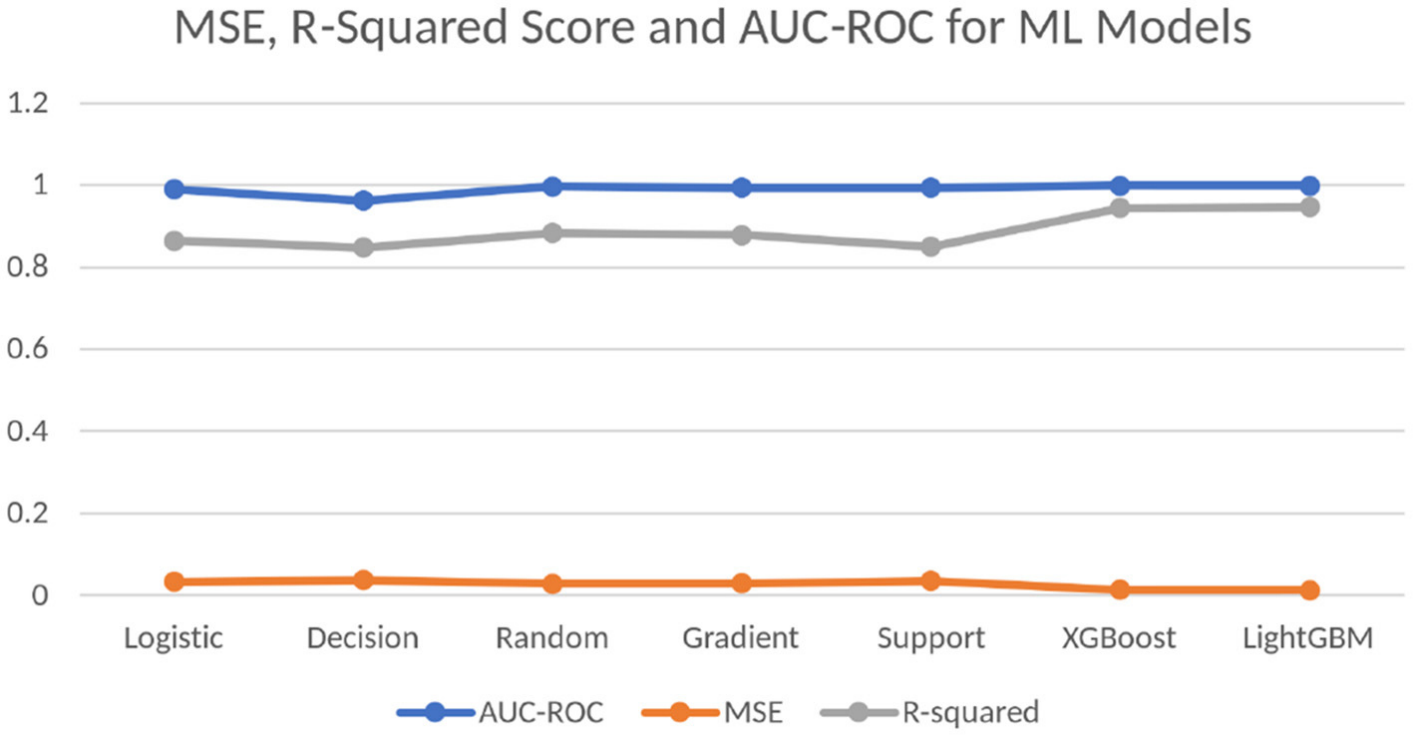
Visualized comparison of the scores of the mean squared error test, *R*-squared test, and AUC-ROC values of the ML models.

The training started with initially taking the default parameter values for the DL models, however, eventually, the values are changed slightly by increasing and decreasing them. A comprehensive log is made to analyze the results of the training quality of models with the different parameters and the best trade-off values to the nearest round-off values are chosen for the parameter values. Also, the “Adam” optimizer is implemented in each model to study the effect of the parameters. The batch size is based on the computing power of the training computer used, whereas the epochs value is decided to handle the probable problem of overfitting during training. Table 4 shows the mean square error (MSE) and *R*-squared scores for the ML models and Figure 2 visualizes Table 4. From the table values, it is deduced that the models are trained well, and the error is reduced. These statistical tests further show that the models have been successful in being properly trained and giving accurate predictions with low to no errors.

**Table 4:** Mean square rrror (MSE), *R*-squared score and area under Roc curve for the machine learning models.

**Classifier**	**MSE**	***R*-squared**	**AUC-ROC**
Logistic regression	0.033806	0.864532	0.989483
Decision tree	0.03798	0.847816	0.962067
Random forest	0.029215	0.882932	0.997036
Gradient boosting	0.030259	0.878761	0.99406
Support vector machine	0.035684	0.857017	0.993934
XGBoost	0.013356	0.946481	0.998583
LightGBM	0.013773	0.944796	0.998485

### 4.5 Feature importance extraction

Finally, after the ML-DL models have been successfully trained and tested using the *k*-folds cross-validation technique, the feature importance extraction is executed. The objective is to derive feature importance solely from the machine learning model that exhibits the highest level of accuracy and training quality (Dara and Tumma, 2018; Khalid et al., 2014). This analysis expects to find the importance of features such as smoking, alcohol, particulate matter, environmental gases, Stroop test and more. This section's findings will help to determine the impact of each component on prediction. This phase is different from the initial feature extraction stage. Initially, the cleaning stage deleted the columns or attributes that would not affect the predictions. However, in this stage, only the most accurate ML model is used, XGBoost, to find how important the feature was using quantitative measurements in terms of importance scores. The most important reason for using only the best ML model is the fact that DL models use automatic feature extraction, thus the importance of features from those readings might not correlate accurately to the existing attributes. This is the penultimate stage of the workflow, whereas, in the ultimate stage, the paper only focuses on statistical testing and validation of the results.

## 5 Experimental results and discussion

**Figure 3:**
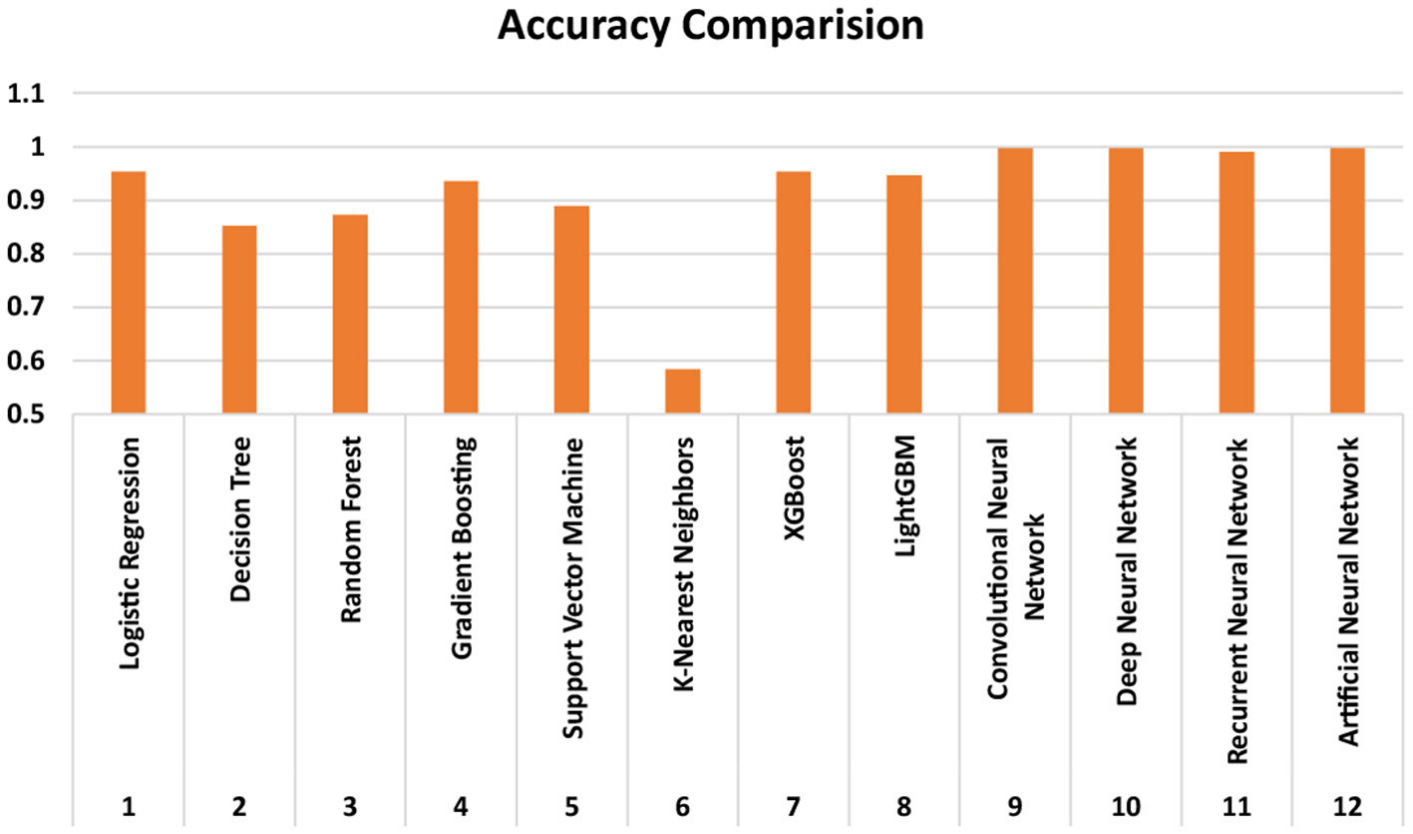
Visualization and comparison of the accuracy, precision, recall and *F*1-scores of the ML and DL models.

The proposed study analyzed the relationship between environmental and lifestyle factors and the prevalence of mental disorders in individuals. The results of the Stroop test are also used to get quantitative support for the predictions, as most of the other results on addiction are self-examined answers to a fixed questionnaire. The occurrences of the number of mental disorders in a person are predicted as above or below the average value of the entire population. The research achieved high accuracy results, post hyperparameter tuning, and *K*-folds technique, as summarized in Table 5 and visualized in Figure 3.

**Table 5:** Comparison of performance measure for different ML And DL models.

**S. No**.	**Classifier**	**Accuracy**	**Precision**	**Recall**	***F*1-score**
**Machine learning models**					
1	Logistic regression	0.953177	0.954377	0.95318	0.953089
2	Decision tree	0.852843	0.853267	0.85284	0.852902
3	Random forest	0.87291	0.87305	0.87291	0.872941
4	Gradient boosting	0.936455	0.936853	0.93646	0.936391
5	Support vector machine	0.889632	0.889885	0.88963	0.889669
6	*K*-nearest neighbors	0.585284	0.586493	0.58528	0.58547
7	XGBoost	0.953177	0.953449	0.95318	0.953142
8	LightGBM	0.946488	0.946747	0.94649	0.946447
**Deep learning models**					
9	DNN1	0.997916	0.999127	0.99649	0.9978
10	DNN2	0.996874	0.999115	0.99442	0.996708
11	DNN3	0.990192	0.995411	0.98409	0.989569
12	DNN4	0.997708	0.999064	0.99613	0.99755

### 5.1 Performance of machine learning and deep learning models

**Figure 4:**
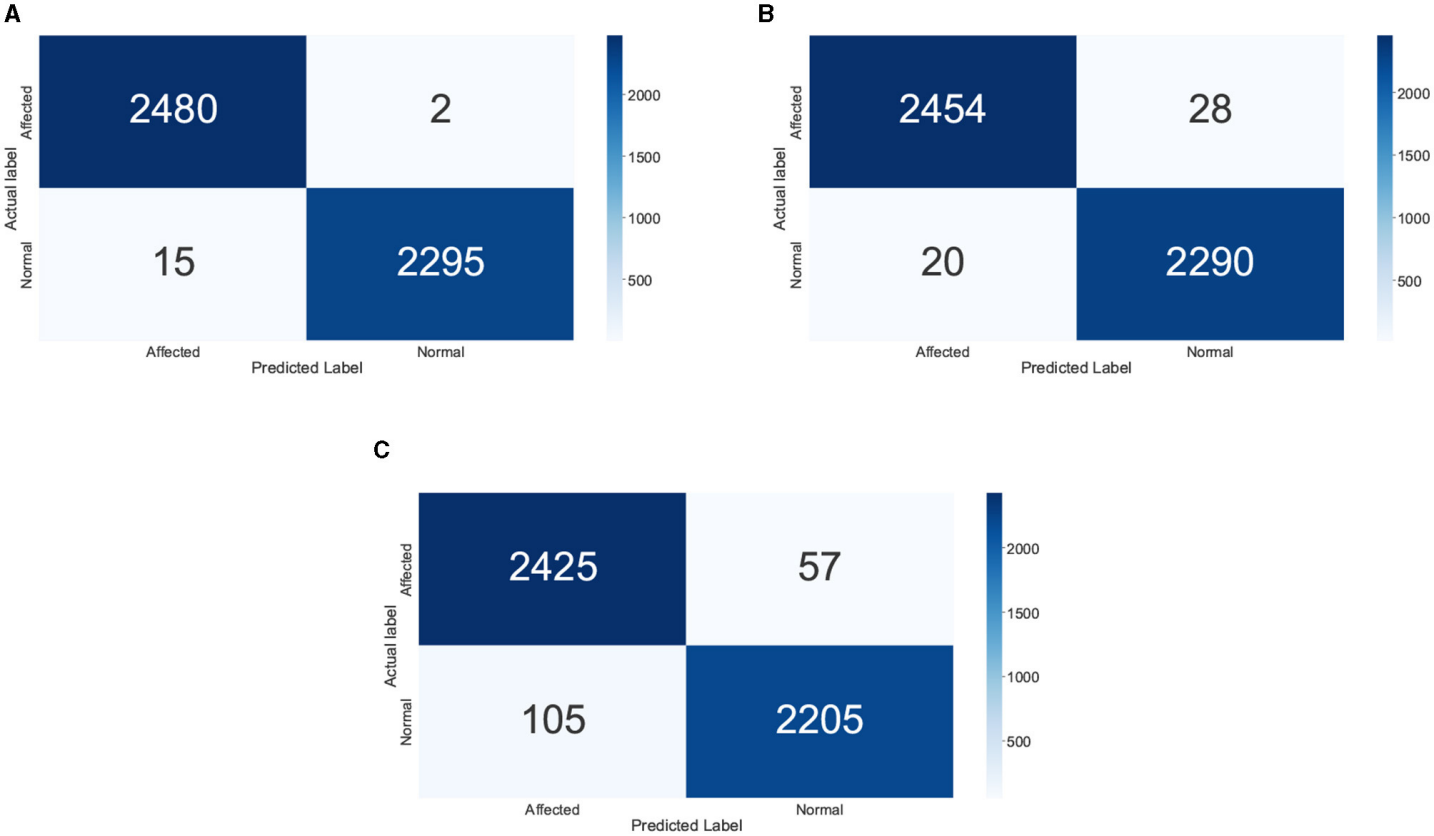
Confusion matrix of the different AI techniques: **(A)** DNN1, **(B)** XG Boost, and **(C)** LR.

In the DL models, DNN1 performed the best with an accuracy of 99.79%, whereas the performance accuracy of DNN4 is also comparable with an accuracy of 99.77%. DNN2 and DNN3 followed next with 99.6 and 99.0% accuracy, respectively. On the other hand, among the machine learning models, the XGBoost and Logistic Regression models yield the best accuracy of 95.31%. However, logistic regression had a slightly higher precision value at 95.43% as compared to XGBoost at 95.34%. The trend in textitF-score is reversed, with XGBoost having a slightly higher value of 95.31% as compared to logistic regression's value of 95.3%. The confusion matrices of the DNN1 model, XGBoost and Logistic Regression are shown in Figure 4. From the results, it is evident that the DNN1 model performed substantially better than the ML models, the same was observed in the accuracy trend. Although XGBoost and Logistic Regression have similar accuracies, the confusion matrices clearly show that XGBoost performs better. This is one of the reasons behind choosing the XGBoost model for extracting the feature importance scores too in the later results.

LightGBM performed closely to these models and offered an accuracy of 94.64%, and the poorest performance is achieved by KNN with an accuracy of merely 58.3%. All other models performed well over 80% accuracy, as depicted in Table 5. These accuracy values about the occurrences of the number of mental disorders in a person are predicted as above or below the average value of the entire population. Thus, no particular disorder will be pinpointed, however, only the prediction of the individual having more or less neurological disorders is found with great accuracy.

### 5.2 Training quality validation and computational matrices of the models

DL models performed better than the ML models in all four parameters; accuracy, precision, recall, and textitF-score. However, DL models take significant computational time in their training and testing. For the 100 epochs run, the DNN1 model took on an average of 129 ms (mili-seconds) per epoch, i.e., 12.9 s for 100 epochs. Using *K*-Fold validation techniques make the model run the 100 epochs 12 times for the number of *K* fixed. Therefore, collectively the DNN1 model took ~155 s to be trained and tested. Similarly, calculated computational times for the DNN3, DNN2 and DNN4 models are 102, 50, and 62 s, respectively, based on their *k*-values. Thus, the research shows that DL models are needed in the research of trends in mental health and substantiate the conclusions from the literature review.

**Figure 5:**
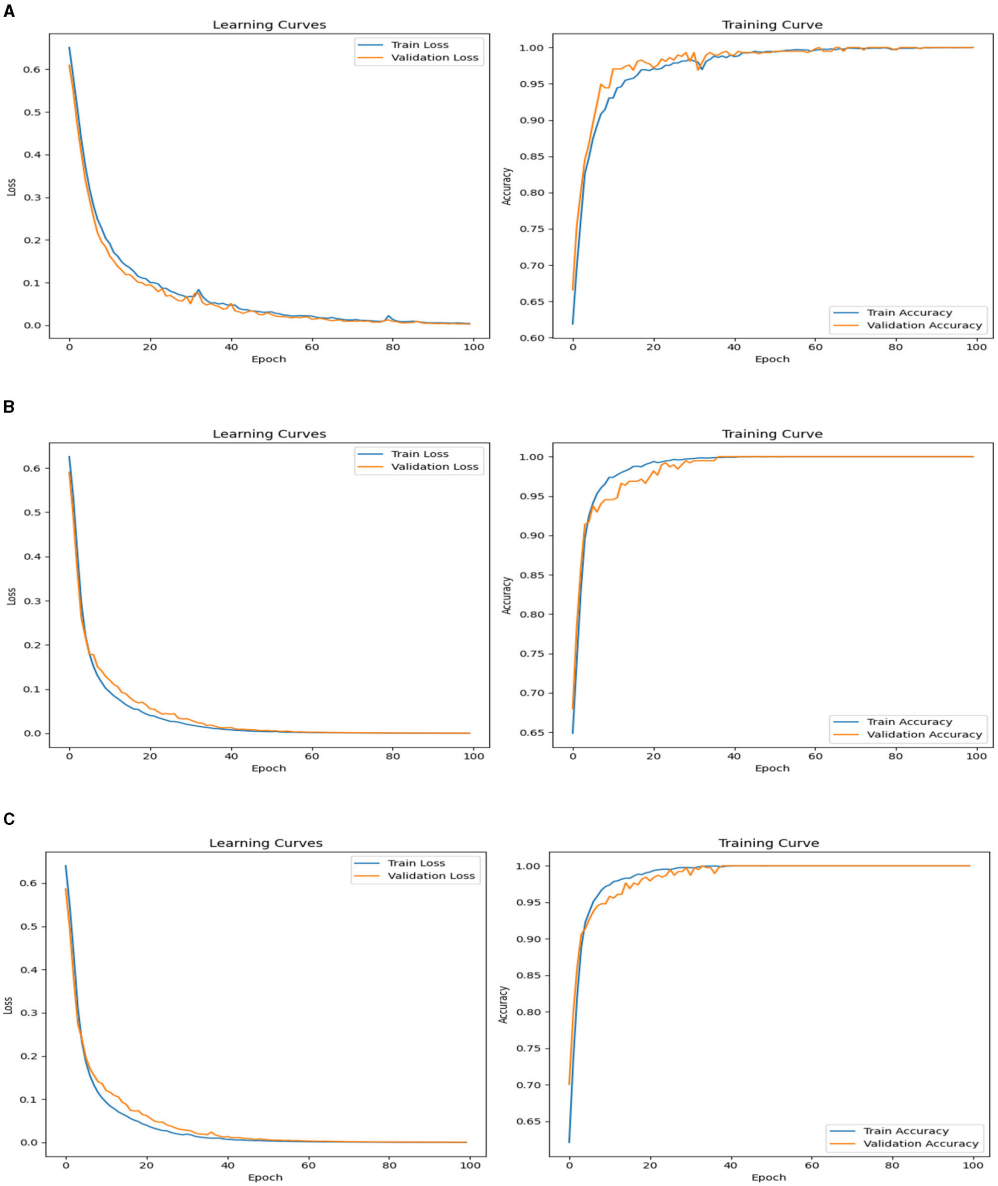
Training loss and accuracy curves for **(A)** DNN1, **(B)** DNN2, and **(C)** DNN4 models.

The learning curve, a mathematical model, shows the relation between loss (*y*-axis) and epochs (*x*-axis). The training curve typically shows training iterations or epochs on the *x*-axis and a performance metric, such as accuracy, loss, or error rate, on the *y*-axis. The two aforementioned curves are presented for the top 3 accurate models; DNN1, DNN2, and DNN4. Figure 5A shows the plot of the learning and training curves of the DNN1 models. It is observed that the training loss and validation loss both converge approximately to 0. Similarly, in the training curve, the train accuracy and validation accuracy converge close to 1. Thus, it is concluded from the two graphs, though few fluctuations are observed, that the model is successfully trained. Similar trends are seen in the learning and training curves of the DNN2 and DNN4 models, shown in Figures 5B, C, respectively. One observation from Figures 5B, C was the high-quality smoothening of the two curves indicating good model training and justification of the accuracies presented.

### 5.3 Feature importance scores and impact of the associated attributes

**Figure 6:**
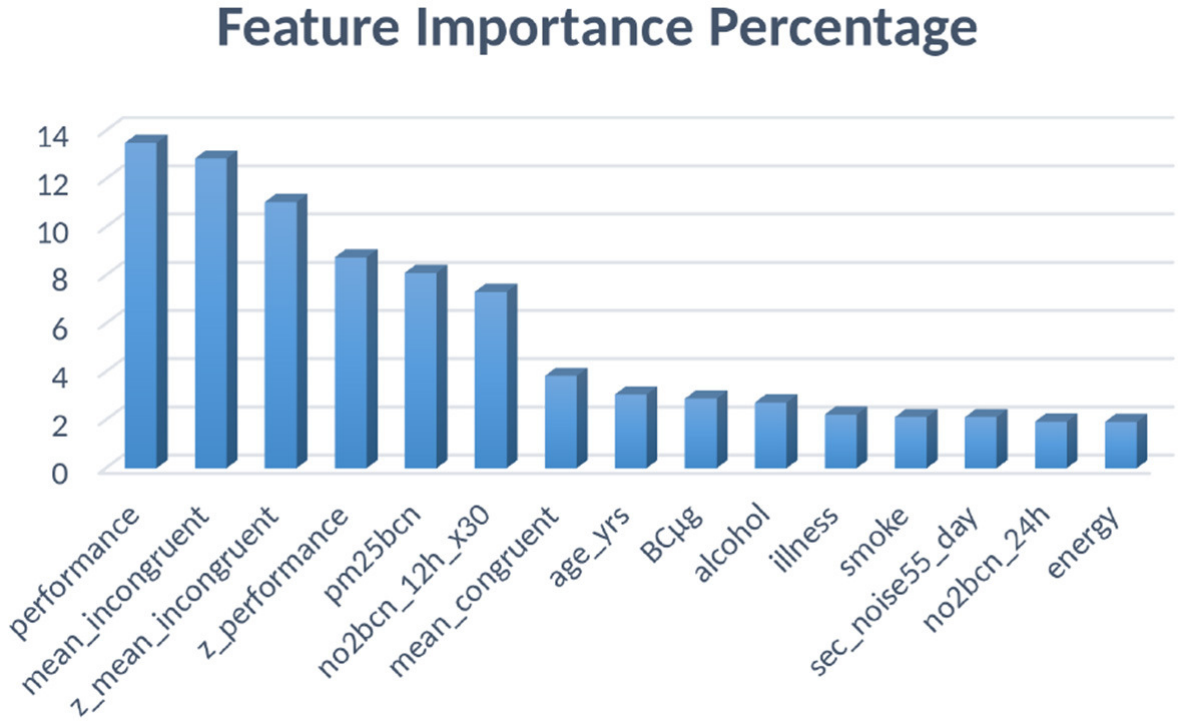
Feature importance percentages for XGBoost classifier.

The research also studied the importance of features on our dataset to be able to analyze the impact of each of the environmental and lifestyle factors in detail. Table 6 gives a comprehensive summary of the best 15 features using the XGBoost classifier, and the percentages of the same have been visualized in Figure 6. From the table, it is seen that the Stroop test performance and its incongruent scores' *z*-values are the most crucial factors in mental health prediction.

**Table 6:** Feature importance using XGBoost classifier.

**Classifier: XGBoost**	**Feature importance**
Performance	0.135
mean_incongruent	0.1285
z_mean_incongruent	0.1104
z_performance	0.0875
pm25bcn	0.0811
no2bcn_12h_x30	0.0732
mean_congruent	0.0383
age_yrs	0.0306
BCμg	0.0289
Alcohol	0.0273
Illness	0.0223
Smoke	0.0212
sec_noise55_day	0.0212
no2bcn_24h	0.0193
Energy	0.0192

**Figure 7:**
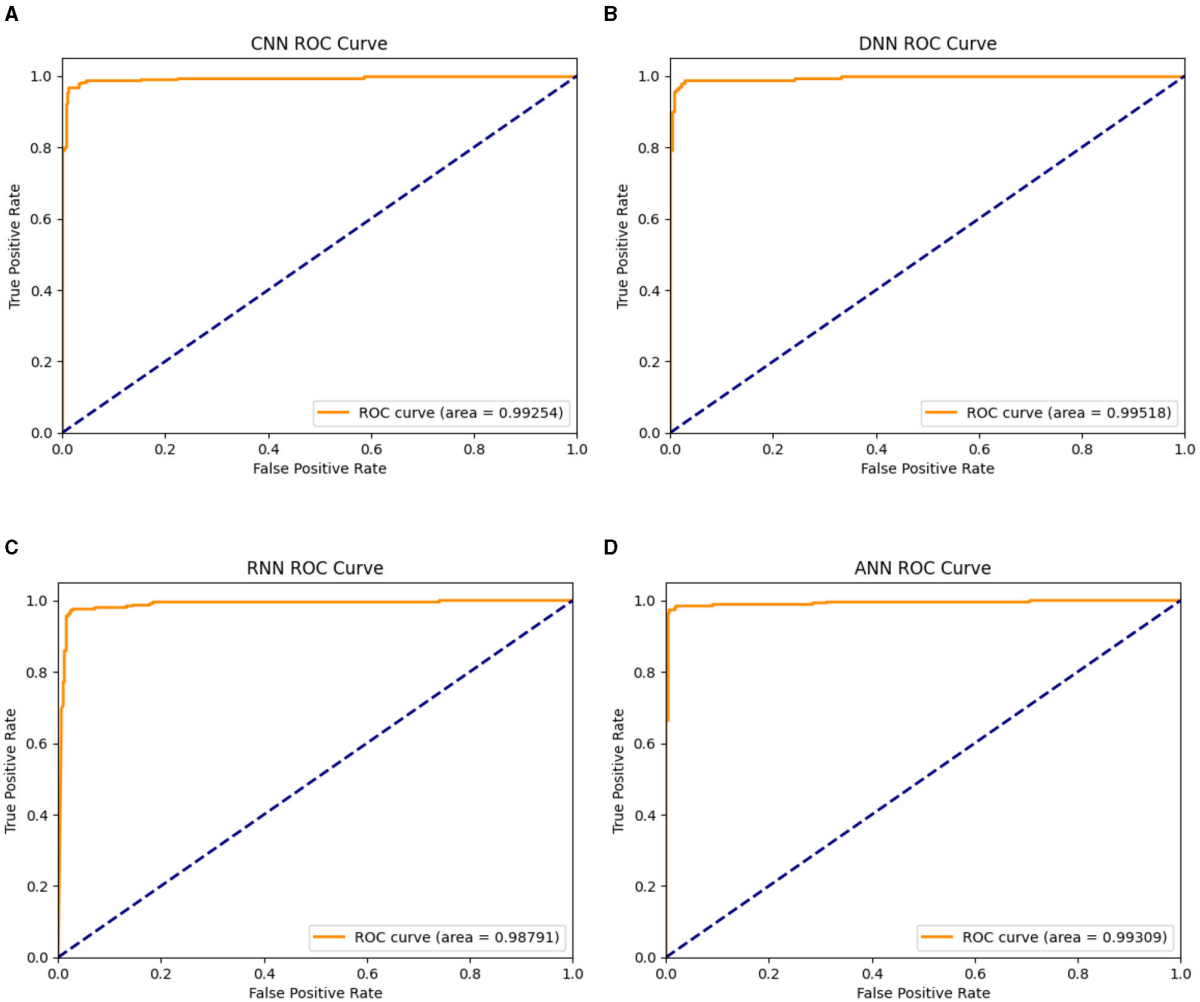
Visualization of the AUC values of the DL models; **(A)** DNN1, **(B)** DNN2, **(C)** DNN3, and **(D)** DNN4.

Stroop test is used specifically for testing mental health and cognitive ability and thus, it was expected to be the maximum, and points toward successful training of the model. The best four features are related to the Stroop test and thus justify the claims made by this research. The next most important column is “pm25bcn”, which represents the value of particulate matter in the air. The next column to this represents *NO*_2_ values. These results clearly show that air pollution is one of the most important factors in predicting mental health. The feature “alcohol” is among the top ten most influential features and represents the effect of lifestyle choices on mental health. One more important method to evaluate the performance of training models is to evaluate the ROC curves and calculate values of the area under the curve (AUC) of the models. The obtained ROC curves of the four DL models are shown in Figure 7. Table 7 reports the results of the AUC values for the four DL models. Table 4 shows the AUC values for the ML models, with XGBoost having a maximum AUC of 0.998583, which supports the results on the model's accuracy. The same has been visualized in Figure 2.

**Table 7:** The area under the ROC curve values (AUC) for the deep learning models.

**Classifier**	**AUC**
DNN1	0.99253
DNN2	0.99518
DNN3	0.9879
DNN4	0.99308

As a result, the findings highlight the significance of their lifestyle choices as well as environmental influences over which they may have no direct control. The use of quantitative testing measures like the Stroop test reveals that quantitative measure results are highly efficient in predicting the mental health of an individual. PM 2.5, black carbon and *NO*_2 are indeed the most impactful environmental factors affecting mental health. Alcohol has a much larger impact on the mental health of an individual as compared to smoking; however, both are influential life choices.

### 5.4 Performance comparison of the proposed framework with state-of-the-art techniques

In this study, authors conducted a comprehensive performance comparison of our proposed method with several existing approaches, as summarized in Table 8. The table provides insights into the effectiveness of different techniques across various literature sources. It is to be noted that this is a first-of-its-kind research on this Cities-Health dataset, therefore, no pre-existing comparison models are reported in Table 8 with the same data. Notably, Mutalib et al. (2021) achieved an accuracy of 88.00% in the context of students in higher education in Kuala Terengganu, while Sabourin et al. (2019) reported an accuracy of 83.33% for Pharm D students at the University of Michigan. The remaining methods proposed in the literature in Hou et al. (2016) and Spyrou et al. (2016) achieved better accuracy of more than 95% compared to the previous literature in Mutalib et al. (2021) and Sabourin et al. (2019). Our proposed method, implemented on the Cities-Health dataset, outperformed these benchmarks with an outstanding accuracy of 99.79%. This superior performance can be attributed to the utilization of 12 innovative techniques tailored to the unique characteristics of the Cities-Health dataset. The results underscore the efficacy of our approach and its potential applicability in diverse scenarios. The previous research (Hou et al., 2016; Spyrou et al., 2016) on different datasets achieved high accuracy with testing 4 and 3 methods, respectively. This research achieves even higher and more accurate results by utilizing 12 different techniques to find out the best method for the predictions and also ensure that no other stand-alone methods can perform better. The use of ensemble techniques has not been done in this research and thus, the complexity of the models must be calculated for each separately. Though 12 models have been trained and tested, in real-world applications, only the best-performing trained model will be deployed with better computational efficiency than the previous methods. Optimizing performance through multi-model training is reliant upon various parameters, including model selection, data volume and quality, problem intricacy, and processing power. Robustness is improved by a variety of models and large amounts of diverse, variable data; however, complex tasks and adequate processing capacity are necessary for efficient implementation. Furthermore, the study also explores other relevant dimensions, such as the number of techniques employed and the specific databases utilized, contributing valuable insights to the field.

**Table 8:** Performance comparison of the proposed method with literature.

**Literature**	**Year**	**Database**	**No. of techniques**	**Accuracy (in %)**
Mutalib et al. (2021)	2021	Students in higher education, Kuala Terengganu	5	88.00
Sabourin et al. (2019)	2019	PharmD students, University of Michigan	4	83.33
Hou et al. (2016)	2016	Students from University	4	96.70
Spyrou et al. (2016)	2016	34 participants	3	96.20
Tutun et al. (2023)	2023	NEPAR-Q and NEPAR-P	2	89.00
Proposed method	2023	Cities-health	12	99.79

## 6 Conclusion

The pilot study of this work resented a way to predict the trend in mental health disorders with the population studied. The work presented quantitative results that support our claim of the effect of various factors on an individual's mental health. Quantitative and qualitative results show that the hypothesis of pollution and addiction affecting the mental health of adults is correct, and steps must be taken to address these serious concerns. The outcome of the work will help to understand the severe effects of the choices they make by using alcohol and cigarettes on their mental health. Future scopes include the development of a dataset that represents all walks of life and all demographics, thus, in turn, training a more generalized AI model to predict mental health occurrence. This study is bound to help doctors and mental health experts predict the occurrence of mental health, especially in cases of a natural disaster or adversity that may alter the environment. However, it's important to perform the experiments on multiple datasets of similar kinds to reach a more sound medical conclusion. Additionally, this research will also support policymakers in understanding the influence of environment and addictions on the mental health of adults. It will help them frame laws that will help better society.

## Data availability statement

Publicly available datasets were analyzed in this study. This data can be found here: Cities-Health Project, Barcelona.

## Ethics statement

Ethical approval was not required for the study involving humans in accordance with the local legislation and institutional requirements. Written informed consent to participate in this study was not required from the participants or the participants' legal guardians/next of kin in accordance with the national legislation and the institutional requirements. Written informed consent was not obtained from the individual(s) for the publication of any potentially identifiable images or data included in this article because the research uses an Open-access dataset in CC copyright laws, thus, the humans in question does not directly relate to the research and their identities are skipped in the dataset since acquisition.

## Author contributions

ND: Conceptualization, Data curation, Formal analysis, Investigation, Methodology, Software, Validation, Visualization, Writing—original draft, Writing—review & editing, Project administration. NS: Conceptualization, Data curation, Formal analysis, Investigation, Methodology, Writing—original draft, Writing—review & editing. MM: Formal analysis, Funding acquisition, Project administration, Resources, Supervision, Writing—review & editing. KP: Formal analysis, Funding acquisition, Project administration, Resources, Supervision, Validation, Writing—review & editing. PK: Formal analysis, Resources, Supervision, Validation, Writing—review & editing. NA: Resources, Supervision, Writing—review & editing. RT: Funding acquisition, Project administration, Resources, Supervision, Writing—review & editing. PP: Formal analysis, Funding acquisition, Project administration, Resources, Supervision, Writing—review & editing. AJP: Formal analysis, Funding acquisition, Project administration, Resources, Supervision, Validation, Writing—original draft, Writing—review & editing.

## Footnotes

^1^Fact Sheets. Accessed on the current date.

^2^Cities-Health. https://www.citieshealth.eu/ (accessed August 1, 2023).
